# DHA Production in *Escherichia coli* by Expressing Reconstituted Key Genes of Polyketide Synthase Pathway from Marine Bacteria

**DOI:** 10.1371/journal.pone.0162861

**Published:** 2016-09-20

**Authors:** Yun-Feng Peng, Wen-Chao Chen, Kang Xiao, Lin Xu, Lian Wang, Xia Wan

**Affiliations:** 1 Key Laboratory of Biology and Genetic Improvement of Oil Crops, Ministry of Agriculture, Oil Crops Research Institute of Chinese Academy of Agricultural Sciences, Wuhan, China; 2 Hubei Key Laboratory of Lipid Chemistry and Nutrition, Wuhan, China; University of Huddersfield, UNITED KINGDOM

## Abstract

The gene encoding phosphopantetheinyl transferase (PPTase), *pfaE*, a component of the polyketide synthase (PKS) pathway, is crucial for the production of docosahexaenoic acid (DHA, 22:6ω3), along with the other *pfa* cluster members *pfaA*, *pfaB*, *pfaC* and *pfaD*. DHA was produced in *Escherichia coli* by co-expressing *pfaABCD* from DHA-producing *Colwellia psychrerythraea* 34H with one of four *pfaE* genes from bacteria producing arachidonic acid (ARA, 20:4ω6), eicosapentaenoic acid (EPA, 20:5ω3) or DHA, respectively. Substitution of the *pfaE* gene from different strain source in *E*. *coli* did not influence the function of the PKS pathway producing DHA, although they led to different DHA yields and fatty acid profiles. This result suggested that the *pfaE* gene could be switchable between these strains for the production of DHA. The DHA production by expressing the reconstituted PKS pathway was also investigated in different *E*. *coli* strains, at different temperatures, or with the treatment of cerulenin. The highest DHA production, 2.2 mg of DHA per gram of dry cell weight or 4.1% of total fatty acids, was obtained by co-expressing *pfaE*(EPA) from the EPA-producing strain *Shewanella baltica* with *pfaABCD* in DH5α. Incubation at low temperature (10–15°C) resulted in higher accumulation of DHA compared to higher temperatures. The addition of cerulenin to the medium increased the proportion of DHA and saturated fatty acids, including C12:0, C14:0 and C16:0, at the expense of monounsaturated fatty acids, including C16:1 and C18:1. Supplementation with 1 mg/L cerulenin resulted in the highest DHA yield of 2.4 mg/L upon co-expression of *pfaE*(DHA) from *C*. *psychrerythraea*.

## Introduction

Very-long-chain polyunsaturated fatty acids (VLCPUFAs) such as eicosapentaenoic acid (EPA, 20:5ω3) and docosahexaenoic acid (DHA, 22:6ω3) are essential to human health and nutrition [1]. VLCPUFAs are synthesized by the conventional elongation and desaturation of existing fatty acids [2–4] or biosynthesized *de novo* by a specialized polyketide synthase (PKS). PKSs have been identified primarily in marine bacteria or eukaryotic *Thraustochytrids* [5–7]. Both a conventional desaturase/elongase and an unconventional PKS pathway for the production of VLCPUFAs have been proposed in *Thraustochytrids* [6]. By contrast, only the PKS pathway has been demonstrated to be responsible for the production of VLCPUFAs in marine bacteria [8, 9].

Among the five *pfa* genes involved in the PKS pathway, only *pfaB* has been studied in detail and appears to determine the type of final product, EPA or DHA [10]. The gene *pfaE* encodes a phosphopantetheinyl transferase (PPTase), which transfers the pantetheine moiety from Coenzyme A to a conserved serine residue on inactive carrier protein to produce active carrier protein [11]. This post-translational modification of carrier protein exists in several multi-enzyme systems, including fatty acid synthases (FAS), nonribosomal polypeptide synthases (NRPSs) and PKS. Based on the diversity of carrier protein sequences and a wide range of substrate spectra, PPTases can be classified into three families: *holo*-acyl carrier protein synthase (AcpS-type PPTase), surfactin phosphopantetheinyl transferase (sfp-type PPTase) and type I integrated PPTase [11–13]. The PPTases that are responsible for VLCPUFA production are probably sfp-type enzymes, based on primary structure [14]. The functions of sfp-type PPTases in the biosynthesis of PKS- and NRPS-derived metabolites in bacteria and plants are well documented [12, 15]. *pfaE* is essential for EPA and DHA production in several marine bacteria, but the differential functions of the diversified *pfaE* genes are unclear. A novel functional *pfa* gene cluster recently discovered in *Aureispira marina* is responsible for the production of arachidonic acid (ARA, 20:4ω6) [16]. Therefore, we were interested in determining if *pfaE* genes from other DHA-, EPA- or ARA-producing strains could play similar roles in VLCPUFA production.

We previously cloned and identified all five *pfa* genes from the DHA-producing marine bacterium *Colwellia psychrerythraea* 34H [17]. The function of *pfaE* is essential for VLCPUFA synthesis [14, 18]. To further investigate the function of the sfp-type PPTase involved in the biosynthesis of VLCPUFAs, we examined whether *pfaE* from DHA-, ARA- or EPA-producing strains could replace the *Colwellia pfaE* gene for DHA production in a heterologous expression system. We expressed the *pfa* gene cluster in two plasmids, one carrying *pfaABCD* and the other carrying the *pfaE* gene from one of four VLCPUFA-producing strains. We demonstrated that the co-expression of any of these four *pfaE* genes with *pfaABCD* in *E*. *coli* resulted in comparable DHA production, suggesting that these genes are exchangeable. Optimized at different temperatures, with or without cerulenin treatment, the reconstituted expression of *pfaABCDE* resulted in as high as 2.2 mg DHA per gram dry cell weight in *E*. *coli*.

## Materials and Methods

### Bacterial strains, plasmids and growth conditions

The cell line *C*. *psychrerythraea* 34H was purchased from American Type Culture Collection (ATCC, BAA-681). The *E*. *coli* strains and plasmids used in this study are listed in Table 1. *C*. *psychrerythraea* 34H was cultured in Marine Broth 2216 (Difco, MI, USA) at 10°C with shaking at 180 rpm. The recombinant *E*. *coli* cells carrying different plasmids were cultivated in Luria–Bertani medium (LB, 1% tryptone, 0.5% yeast extract, and 1% NaCl) supplemented with 100 mg/L ampicillin or 34 mg/L chloramphenicol corresponding to the vectors used. Half concentrations of antibiotics were used when two compatible vectors were co-expressed.

**Table 1:** Strains and plasmids used in this study.

Strain or plasmid	Description	Source
**Strain**		
*E*. *coli* DH5α	F^-^, φ80d*lacZ* ΔM15, Δ(*lac*ZYA -*arg*F )U169, *deo*R, *rec*A1, *end*A1, *hsd*R17 (rK^-^, mK^+^), *pho*A, *sup*E44, *λ*^-**^, *thi*-1, *gyr*A96, *rel*A1	Takara Bio
*E*. *coli* JM109	*rec*A1, *end*A1, *gyr*A96,* thi*-1, *hsd*R17(rk^-^mk^+^),* e*14^-**^*(mcr*A^-^* )sup*E44, *rel*A1, Δ(*lac*-*pro*AB )/F' [*tra*D36, *pro*AB^+^, *lac*I,* lac*ZΔM15]	Takara Bio
*E*. *coli* HB101	*sup*E44, Δ(*mcr*C-*mr*r),* rec*A13, *ara*-14, *pro*A2, *lac*Y1, *gal*K2,* rps*L20, *xyl*-5, *mtl*-1, *leu*B6,* thi*-1	Takara Bio
*E*. *coli* HST08	F^-^, *end*A1, *sup*E44, *thi*-1,* rec*A1, *rel*A1, *gyr*A96,* pho*A, Φ80d *lac*Z, ΔM15, Δ(*lac*ZYA - *arg*F)U169, Δ(*mrr* - *hsd*RMS - *mcr*BC), *Δmcr*A, *λ*^-**^	Takara Bio
**Plasmid**		
pColdI	Cold-shock expression vector, Amp	Takara Bio
pColdI-*pfaABCD*	pColdI carrying *pfaA*, *pfaB*, *pfaC*, *pfaD* from *C*. *psychrerythraea*	This study
pSTV28	Low-copy-number cloning vector, Cm^r^	Takara Bio
pSTV28::*pfa*E(DHA)	pSTV28 carrying *pfaE* from *C*. *psychrerythraea*	This study
pSTV28::*pfa*E(DHA-M)	pSTV28 carrying *pfaE* from *Moritella marina* MP-1	This study
pSTV28::*pfa*E(EPA)	pSTV28 carrying *pfaE* from *S*. *baltica* OS678	This study
pSTV28::*pfa*E(ARA)	pSTV28 carrying *pfaE* from *Aureispira marina*	This study

### Cloning of *pfa* genes from *C*. *psychrerythraea* and three other marine bacteria

*C*. *psychrerythraea* genomic DNA was extracted from cells using a Bacteria Gen DNA Extraction kit (CWBIO, China) according to the manufacturer’s instructions. Four *pfa* genes, *pfaA*, *pfaB*, *pfaC* and *pfaD*, were cloned into pColdI (Takara Bio, Dalian, China) in our previous study [17]. PCR was performed using Prime STAR GXL DNA Polymerase (Takara Bio) with the forward primer pfaA-F containing an *EcoR*I restriction site and the reverse primer pfaD-R containing a *Sal*I restriction site (S1 Table). The resulting DNA fragment of approximately 20 kb was double digested with *EcoR*I and *Sal*I and then subcloned into the pColdI vector to generate the plasmid pColdI-*pfaABCD*.

The *C*. *psychrerythraea* 34H *pfaE* gene (*CPS_RS13895*, http://www.ncbi.nlm.nih.gov/) was amplified from genomic DNA with the specific primers pfaE-1F and pfaE-1R (S1 Table). Three other *pfaE* genes from DHA-producing *Moritella marina* MP-1 (Accession AB262366), EPA-producing *Shewanella baltica* OS678 (Accession CP002383) and ARA-producing *Aureispira marina* (Accession AB980240) were synthesized according to the published sequences flanked by the restriction sites *Kpn*I and *Bam*HI. The four *pfaE* genes were each double-digested and subcloned into pSTV28 to generate the plasmids pSTV28::*pfaE*(DHA), pSTV28::*pfaE*(DHA-M), pSTV28::*pfaE*(EPA) and pSTV28::*pfaE*(ARA), respectively (Table 1).

### Heterologous co-expression of the five *pfa* genes in *E*. *coli*

The constructed vectors harboring *pfaABCD* and one of four different individual *pfaE* genes were introduced into *E*. *coli* cells by electroporation. The empty vector pColdI or the vector pColdI-*pfaABCD* alone, without the *pfaE* gene, was used as a control. Primer pairs, including pfaB-F/pfaB-R and pfaE-1F/pfaE-1R, were used for colony PCR to confirm positive clones (S1 Table). A single colony was cultivated in LB medium supplemented with 17 mg/L chloramphenicol and 50 mg/L ampicillin. Approximately 400 μL of pre-cultured cells was transferred to 40 mL of fresh LB medium with the corresponding antibiotics. After incubation for 4 h at 30°C, isopropyl β-D-1-thiogalactopyranoside (IPTG) was added to the culture at a final concentration of 1 mM. The culture was then incubated at 15°C for an additional 48 h. The cells were collected and freeze-dried overnight.

To compare the effect of culture temperature on DHA levels in the recombinant *E*. *coli* DH5α, the cells were incubated at 10, 15 and 20°C, respectively. The effect of cerulenin was also investigated by adding it to LB medium at various concentrations (0, 0.5, 1 and 2 mg/L) from the ethanol stock solution prior to cultivation.

### Analysis of DHA production and the fatty acid profile

One hundred μg of heneicosanoic acid (21:0) was used as an internal standard (Larodan, Sweden) for each sample. Fatty acid methyl esters (FAMEs) from the cells were prepared by the direct acidic trans-methylation method [19]. FAMEs were analyzed by gas chromatography (7890A, Agilent Technologies, USA) equipped with a flame ionization detector (FID) and an HP-FFAP capillary column (30 m × 250 μm × 0.25 μm). High-purity nitrogen was used as the carrier gas. FAMEs were identified by comparison of retention times with those of authentic standards. The relative amount of FAME was quantified by comparing each peak area to the standard GLC 411 (Nu-Chek, USA). For detection of hydroxyl fatty acids, the FAMEs were further derivatized by trimethylchlorosilane as reported to produce trimethylsilyl (TMS) derivatives [19]. Picolinyl esters were prepared for characterization of cyclopropane fatty acid (CPFA) [20]. GC-MS analysis was performed according to Zhou et al [19].

### Detection of the transcriptional level of *pfa* genes in recombinant cells by quantitative real-time RT-PCR (qRT-PCR)

Total RNA was isolated from recombinant cells using an RNeasy Mini Kit (Qiagen) according to the manufacturer’s instructions. First-strand cDNA was synthesized using a Prime Script RT reagent kit with gDNA eraser (Takara). Two microliters of RT product was used as a template for qRT-PCR with the SYBR *Premix ExTaq* II kit. The qRT-PCR mixture (25 μL) contained 2 μL of cDNA, 0.4 μM each gene-specific primer and SYBR *Premix Ex Taq* II (Takara). qRT-PCR was performed as follows: denaturation at 94°C for 4 min, 40 cycles of 30 sec at 94°C, 30 sec at 55°C, and 30 sec at 72°C, and a melting cycle from 55°C to 94°C to check for amplification specificity. The constitutively expressed *E*. *coli* 16S ribosomal RNA gene was used as a reference. The relative abundance of mRNAs was normalized against the levels of the internal control. The primers are listed in S1 Table. All experiments were performed in triplicate.

## Results and Discussion

### Production of DHA in various *E*. *coli* strains

**Fig 1:**
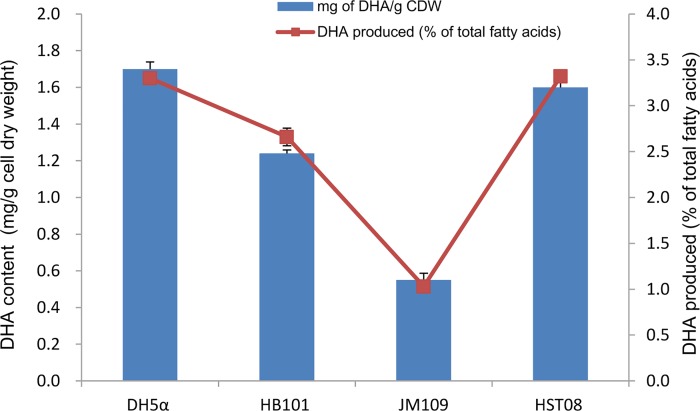
Production of DHA in four *E*. *coli* strains by heterologous expression of *Colwellia pfaABCD* and *pfaE*. The cells were cultured at 15°C with shaking at 180 rpm. The quantification of DHA from each sample was repeated in triplicate (n = 3).

The *Colwellia pfa* gene cluster was identified as responsible for DHA production in our previous study [17]. To further screen the best *E*. *coli* host for the production of DHA, in the present study, the *Colwellia pfa* gene cluster containing all five *pfa* genes was transformed into *E*. *coli* DH5α, JM109, HB101 and HST08. Expression of *Colwellia pfa* genes in the different hosts was induced by the addition of IPTG. As shown in Fig 1, DH5α and HST08 were more suitable hosts for DHA accumulation than JM109 and HB101. The DHA content in the recombinant DH5α and TS08 cells was approximately 1.7 and 1.6 mg/g cell dry weight (CDW), respectively. In addition, DHA accumulated up to 3.3% of total fatty acids in both DH5α and HST08 cells (Fig 1). By contrast, expression of *Colwellia pfa* genes in JM109 cells resulted in the lowest DHA content, 0.6 mg/g CDW (Fig 1). Therefore, DH5α was chosen as the *E*. *coli* host for the following experiments. The production of DHA in *E*. *coli* strains has also been achieved by heterologous expression of the *pfa* gene cluster from *M*. *marina* MP-1 (5.2% of total fatty acids) and *S*. *baltica* MAC1 (0.4% of total fatty acids) [18, 21]. In addition, expression of the *Shewanella pfa* gene cluster in food-grade *Lactococcus lactis* successfully produced both DHA (1.35 mg/g CDW) and EPA (0.12 mg/g DCW) [22]. Compared to the published data, the DHA content of recombinant *E*. *coli* harboring the *Colwellia pfa* gene cluster was not the highest in these growth conditions.

### Co-expression of different individual *pfaE* genes with *Colwellia pfaABCD*

**Fig 2:**
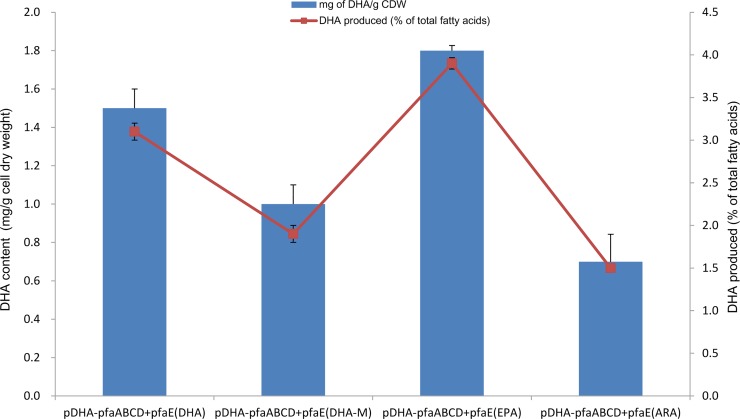
Complementation of the function of *Colwellia pfaABCD* by four different individual *pfaE* genes: *pfaE*(DHA), *pfaE* from *C*. *psychrerythraea*; *pfaE*(DHA-M), *pfaE* from *M*. *marina* MP-1; *pfaE*(EPA), *pfaE* from *S*. *baltica* OS678; and *pfaE*(ARA), *pfaE* from *Aureispira marina*. The cells were cultured at 15°C with shaking at 180 rpm. The quantification of DHA from each sample was repeated in triplicate (n = 3).

Four representative *pfaE* genes (*pfaE*(DHA), *pfaE*(DHA-M), *pfaE*(EPA), and *pfaE*(ARA)) were individually co-expressed with pColdI-*pfaABCD* in DH5α qRT-PCR analysis demonstrated that *pfaA*, *pfaB*, *pfaC*, *pfaD* and a different *pfaE* were differentially expressed at the transcriptional level in the recombinant cells (S1 Fig). DHA was detected by GC in all recombinant DH5α cells after IPTG induction (Fig 2). These results confirmed that substitution of the *pfaE* genes from other long chain PUFA producing strains did not influence the function of the *Colwellia* PKS pathway producing DHA, although different DHA yields and fatty acid profiles were obtained. The sfp-type PPTase-encoding gene *entD* from *E*. *coli* also complements *pfaE* deficiency in EPA production [23]. The EntD-like PPTase was thought to be involved in polyketide or nonribosomal peptide synthesis but not VLCPUFA formation. Therefore, we speculate that not only different *pfaE* genes but also other sfp-type PPTase genes might participate in the synthesis of VLCPUFAs.

DHA levels were influenced by the expression of different *pfaE* genes. Under identical conditions, recombinant cells harboring *pfaE*(DHA) or *pfaE*(EPA) accumulated up to 3.3–3.7% of total fatty acids as DHA, whereas *pfaE*(ARA) only resulted in 1.7% DHA (Fig 2), suggesting that *pfaE* was responsible for the different DHA titers.

**Fig 3:**
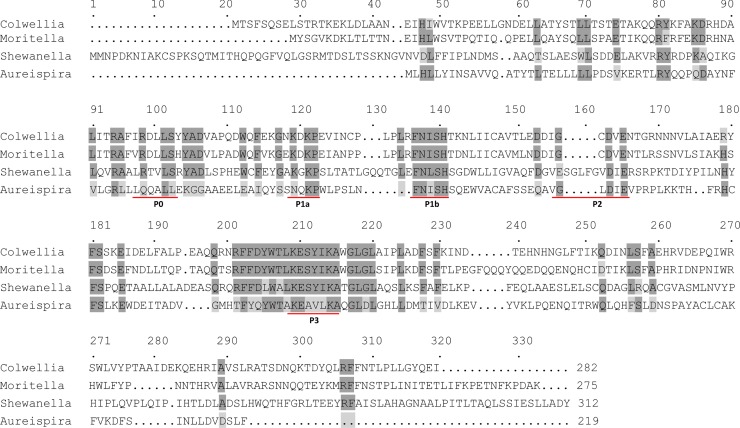
Sequence alignments of the four PfaEs used in this study. *Colwellia*, *C*. *psychrerythraea* 34H; *Moritella*, *M*. *marina* MP-1; *Shewanella*, *S*. *baltica* OS678; and *Aureispira*, *A*. *marina*. Identical amino acid residues are in black, and similar amino acids are in gray. Five putative conserved motifs are marked as P0, P1a, P1b, P2 and P3.

Alignment of the four PfaE protein sequences revealed the existence of P1a, P1b, P2, and P3 domains in these sequences (Fig 3). These conserved amino acid domains were defined previously [11, 14]. The P0 domain was thought to be conserved only in PPTases involved in VLCPUFA production [14]. However, our results indicated that this domain is not conserved among these four PfaE sequences (Fig 3). In addition, the overall sequence similarity was relatively low among these four PfaEs. PfaE(ARA) exhibited the lowest similarity (only 13.4–14.3%) to the other three PfaE sequences, and PfaE(EPA) had a much longer N-terminal sequence compared to the other PfaE sequences (Fig 3). By contrast, PfaE(ARA) had shorter sequences at the N- and C-termini. The low amount of DHA produced by co-expression of *pfaE*(ARA) compared to the high levels of DHA obtained by co-expression of *pfaE*(EPA) (Fig 2) suggest that the N-terminal PfaE(EPA) sequence might be important for the efficiency of transferring the pantetheine moiety to the conserved serine residue on the inactive carrier protein. In conclusion, despite the variations among the different *pfaE* genes, their expression could lead to DHA synthesis.

Several enzymatic domains of marine bacterial PKS have been identified, including ketosynthase (KS), acyl transferase (AT), dehydratase (DH), enoylreductase (ER), ketoreductase (KR), chain-length factor (CLF) and six acyl carrier proteins (ACPs) (EPA-producing *Shewanella* sp.) or five ACPs (DHA-producing *M*. *marina* or *C*. *psychrerythraea*). ACP contains a conserved serine residue that may be the target of post-translational modification by PfaE. Each ACP provides a free thiol for tethering the starter and extender units during the synthesis of fatty acids [11]. The number of ACPs controls the VLCPUFA titer [24]. Because the various *pfaE* genes had different effects on DHA production but the number of ACPs remained the same, we speculated that *pfaE* itself could alter DHA production. This result sheds light on potential for optimizing *pfaE* for improved VLCPUFA yields.

### Effects of temperature on DHA production by recombinant DH5α

**Fig 4:**
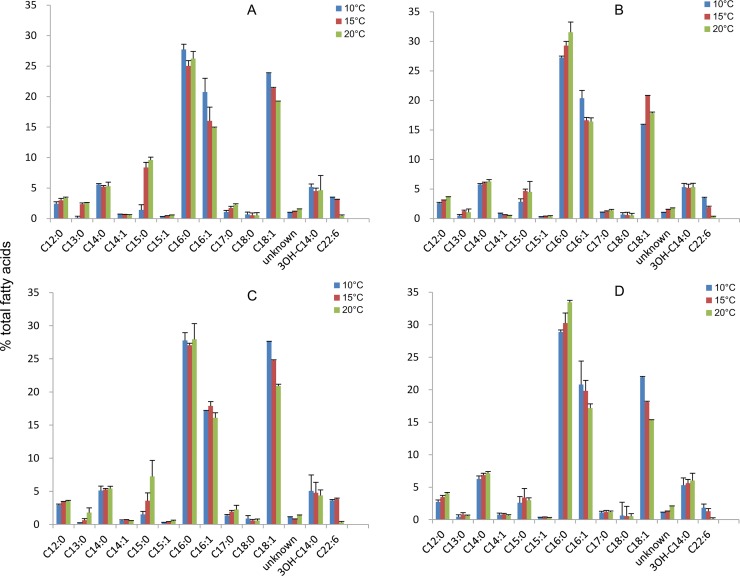
Effect of temperature on the fatty acid profiles of recombinant cells harboring pColdI-*pfaABCD* and different *pfaE* genes. (A) DH5α harboring pColdI-*pfaABCD* and *pfaE*(DHA); (B) DH5α harboring pColdI-*pfaABCD* and *pfaE*(DHA-M); (C) DH5α harboring pColdI-*pfaABCD* and *pfaE*(EPA); (D) DH5α harboring pColdI-*pfaABCD* and *pfaE*(ARA). The quantification of each fatty acid from samples was repeated in triplicate (n = 3).

VLCPUFAs are mainly produced by a narrow subset of marine γ-proteobacteria or marine algae [25,26] from cold environments. Therefore, temperature is considered to be important for VLCPUFA production. Generally, a low temperature (<30°C) is beneficial for VLCPUFA production. In this study, the effect of temperature on DHA production by recombinant DH5α harboring pColdI-*pfaABCD* and different *pfaE* genes was investigated. As shown in Table 2, DHA accumulation increased when the cells were cultured at 10 or 15°C compared to 20°C for all *pfaE* genes. The highest DHA content and yield were achieved by cells harboring *pfaE*(EPA) cultured at 10°C: up to 2.2 mg/g CDW and 1.4 mg/L. The lowest DHA yield, 0.7 mg/L, was achieved by recombinant cells harboring *pfaE*(ARA) cultured at 15°C. However, incubation at 20°C or higher led to decreases in DHA content and yield in all recombinant cells (Table 2). Bacterial DHA is mainly present as a membrane component with uncertain function [27]. Elevated temperature probably reduces membrane fluidity and thus prevents the incorporation of DHA into the membrane. The fatty acid profiles of the recombinant DH5α cells were also investigated. The GC results revealed an increase in the saturated fatty acids (SFA) C15:0 and C16:0 at the expense of the monounsaturated fatty acids (MUFAs) C16:1 and C18:1 in all recombinant cells as the temperature increased (Fig 4). It should be pointed out that the acidic methylation of fatty acids we used in this study would cause some degradation of CPFA, 9,10-methyl-C16:0 existed in host *E*. *coli* strain (S2 Fig), thus the host CPFA was not included in the fatty acid profile. The 9,10-methyl-C16:0 was structurally confirmed by 3-pyridylcarbinyl (picolinyl) ester (S3 Fig). We proposed that the recombinant cells adjust their fatty acid composition in response to thermal fluctuations to allow the cells to regulate membrane fluidity, consistent with observations in wild-type *E*. *coli* [28]. GC-MS confirmed the existence of multiple peaks of C16:1 and C18:1 at close retention time (S4 Fig).

**Table 2:** Effect of temperature on DHA production by recombinant DH5α harboring pColdI-*pfaABCD* and different *pfaE* genes.

Temperature (°C)	DHA (% of total fatty acid)	DHA content (mg/g DCW)	Biomass (g/L)	DHA yield (mg/L)
**pColdI-*pfaABCD*+pSTV28::*pfa*E(DHA)**				
10	3.4 ±0.4	1.8±0.1	0.7±0.0	1.2±0.0
15	3.1±0.1	1.5±0.2	0.9±0.0	1.4±0.2
20	0.5±0.2	0.2±0.1	1.4±0.0	0.3±0.1
**pColdI-*pfaABCD*+pSTV28::*pfa*E(DHA-M)**				
10	3.5±0.3	1.6±0.3	0.8±0.0	1.3±0.3
15	1.9±0.1	1.0±0.1	1.0±0.0	1.0±0.1
20	0.3±0.2	0.1±0.1	1.2±0.0	0.1±0.1
**pColdI-*pfaABCD*+pSTV28::*pfa*E(EPA)**				
10	4.1±0.5	2.2±0.4	0.7±0.0	1.4±0.3
15	3.9±0.1	1.8±0.0	0.8±0.0	1.4±0.1
20	0.3±0.1	0.1±0.1	1.1±0.0	0.2±0.1
**pColdI-*pfaABCD*+pSTV28::*pfa*E(ARA)**				
10	1.8±0.4	1.1±0.3	0.9±0.1	0.9±0.2
15	1.5±0.4	0.7±0.3	1.0±0.1	0.7±0.2
20	0.1±0.2	0.1±0.1	1.2±0.1	0.1±0.1

Cells were cultured at different temperature with constant shaking at 180 rpm. The quantification of DHA from each sample was repeated in triplicate (n = 3).

There were also trace amounts of 3OH-C13:0 and 3OH-C15:0 as well as significant amount of 3OH-C14:0 detected in *E*. *coli* cells (S4 Fig). The corresponding hydroxyl FAME peaks were disappeared after trimethylchlorosilane modification [19], and new TMS-derivatives were appeared (S4 Fig). The structure of these hydroxyl FAMEs were further confirmed by GC-MS, as 3OH-C13:0, 3OH-C14:0 and 3OH-C15:0 (S5 Fig). 3-Hydroxy fatty acids were presented in out membrane lipopolysaccharides of many gram-negative bacteria including *E*. *coli* [29,30]. The tested growth temperature had no obvious effect on 3-hydroxy fatty acids (Fig 4).

### Effects of cerulenin on DHA production by recombinant *E*. *coli*

Cerulenin ([2R,3S]-2,3-epoxy-4-oxo-7,10-trans,tran​s-dodecadienamide) inhibits fatty acid synthesis by irreversibly binding β–ketoacyl-ACP synthase I and II [31]. The addition of cerulenin increases the content of DHA while decreasing MUFAs in several marine bacteria [27,32]. The effect of cerulenin on the production of DHA in recombinant *E*. *coli* was investigated at a cultivation temperature of 15°C due to the relatively higher growth rate and DHA production. As shown in Table 3, both DHA content and DHA yield were enhanced by the addition of cerulenin. The highest DHA content and yield were both achieved in DH5α cells harboring pColdI-*pfaABCD* and *pfaE*(DHA), 3.4 mg/g CDW and 2.4 mg/L, respectively. However, cerulenin at concentrations higher than 1 mg/L slightly inhibited the growth of recombinant cells (Table 3). The recombinant cells did not grow well in the presence of 3 mg/L cerulenin or higher concentrations. In our previous study, 12 mg/L cerulenin was applied to *C*. *psychrerythraea* without obvious inhibition of the growth rate [17]. These results suggest that the recombinant *E*. *coli* cells are more sensitive to cerulenin treatment.

**Table 3:** Effect of cerulenin on DHA production by recombinant DH5α harboring pColdI-*pfaABCD* and different *pfaE*.

Cerulenin treatment (mg/L)	DHA (% of total fatty acid)	DHA content (mg/g DCW)	Biomass (g/L)	DHA yield (mg/L)
**pColdI-*pfaABCD*+pSTV28::*pfa*E(DHA)**				
0	3.3±0.1	1.7±0.1	1.1±0.0	1.9±0.1
0.5	4.7±0.2	2.0±0.1	1.0±0.0	2.0±0.1
1	5.2±0.3	2.7±0.2	0.9±0.1	2.4±0.1
2	7.0±0.3	3.4±0.1	0.7±0.1	2.4±0.1
**pColdI-*pfaABCD*+pSTV28::*pfa*E(DHA-M)**				
0	1.7±0.1	0.9±0.1	1.2±0.0	1.1±0.1
0.5	2.6±0.2	1.1±0.2	1.1±0.0	1.2±0.2
1	3.2±0.3	1.5±0.2	1.0±0.0	1.5±0.2
2	4.7±0.1	2.1±0.1	0.8±0.0	1.7±0.1
**pColdI-*pfaABCD*+pSTV28::*pfa*E(EPA)**				
0	3.7±0.0	1.3±0.1	1.0±0.1	1.3±0.1
0.5	4.2±0.4	1.8±0.2	0.8±0.1	1.4±0.1
1	6.0±0.1	2.5±0.0	0.6±0.0	1.5±0.0
2	4.5±0.2	2.0±0.1	0.6±0.0	1.2±0.1
**pColdI-*pfaABCD*+pSTV28::*pfa*E(ARA)**				
0	1.9±0.2	1.1±0.0	1.2±0.1	1.3±0.1
0.5	3.2±0.2	1.7±0.3	1.0±0.0	1.8±0.3
1	2.3±0.2	1.34±0.2	1.0±0.1	1.4±0.1
2	2.1±0.1	1.2±0.1	0.8±0.0	1.0±0.1

Cells were cultured at 15°C with shaking at 180 rpm. The quantification of DHA from each sample was repeated in triplicate (n = 3).

**Fig 5:**
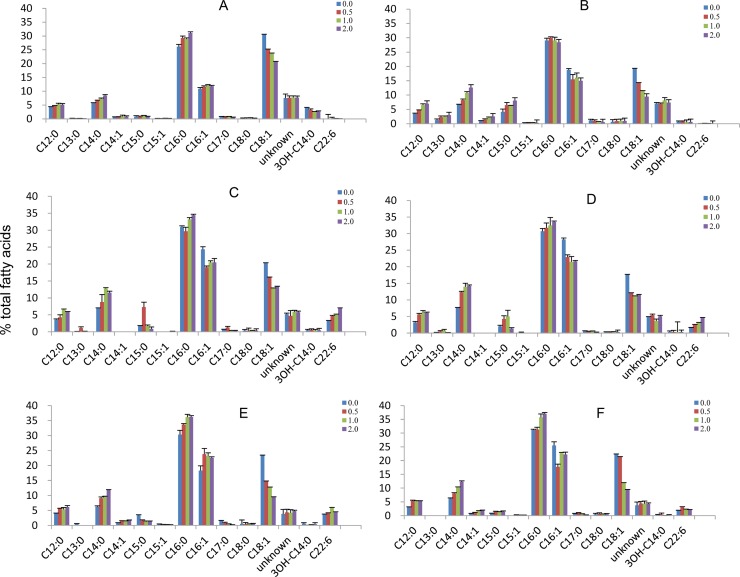
Effect of cerulenin on the fatty acid profile of recombinant cells harboring pColdI-*pfaABCD* and different *pfaE* genes. (A) DH5α harboring empty vector pColdI; (B) DH5α harboring pColdI-*pfaABCD*; (C) DH5α harboring pColdI-*pfaABCD* and *pfaE*(DHA); (D) DH5α harboring pColdI-*pfaABCD* and *pfaE*(DHA-M); (E) DH5α harboring pColdI-*pfaABCD* and *pfaE*(EPA); (F) DH5α harboring pColdI-*pfaABCD* and *pfaE*(ARA). The cells were cultured at 15°C with shaking at 180 rpm. The final concentrations of added cerulenin were 0, 0.5, 1.0 or 2.0 mg/L, as shown in the legend. The quantification of each fatty acid from samples was repeated in triplicate (n = 3).

The fatty acid profiles of different recombinant cells were also analyzed. An increase in SFAs, including C12:0, C14:0 and C16:0, in all recombinant cells was repeatedly observed (Fig 5). By contrast, the proportion of C18:1 was obviously reduced. The proportion of C16:1 was also lower, except in the combination of pColdI-*pfaABCD* and *pfaE*(EPA) (Fig 5E), in which it was higher. In general, FAS and PKS utilize the same substrates, malonyl-CoA and acetyl-CoA, to synthesize various fatty acids [9]. Because cerulenin inhibits FAS activity and enhances DHA production, the fatty acid profile was expected to differ. MUFAs, including C16:1 and C18:1, are lower after treatment with cerulenin due to the inhibition of FabB [31]. The pColdI-*pfaABCD* and *pfaE*(EPA) combination exhibited no significant change in C16:1 at the higher culturing temperature (Fig 4C), in contrast to other combinations, in which the proportions of C16:1 decreased. These results suggest that the longer N-terminal sequence of *pfaE*(EPA) might influence the process of fatty acid synthesis.

Furthermore, the expression of *Colwellia pfaABCD* alone without *pfaE* (Fig 5B) resulted in a higher proportion of C16:1 at the expense of C18:1 compared to cells with empty vector only (Fig 5A), regardless of the amount of cerulenin added. Co-expression of the four individual *pfaE* genes further increased C16:1 but did not further decrease C18:1. These results demonstrate that the expression of *Colwellia pfaABCD* modifies the fatty acid profile, even though DHA was not yet synthesized. The results also suggested that *Colwellia pfaABCD* plays a role in altering the fatty acid profile. When *Colwellia pfaABCD* was co-expressed with one of the four *pfaE* genes, C16:1 and DHA levels were further increased, whereas there were no further changes in the C18:1 level. This further demonstrated the essential function of *pfaE* in producing DHA in *E*. *coli* recombinant cells.

In conclusion, reconstituted expression of the phosphopantetheinyl transferase gene *pfaE* from different VLCPUFA-producing bacteria with other members of the *pfa* gene cluster of *C*. *psychrerythraea* 34H in *E*. *coli* cells consistently resulted in DHA production, although at slightly variable levels. These results suggested that sfp-type PPTases was essential for VLCPUFA production. This also suggested that *C*. *psychrerythraea pfaE* was switchable with different gene sources, providing a potential target for direct evolutionary mutagenesis for higher DHA production. Lower growth temperature and cerulenin treatment for reconstituted *E*. *coli* cells significantly increased the DHA production.

## Supporting Information

S1 Fig Quantitative real-time RT-PCR (qRT-PCR) to detect the transcriptional levels of the *pfa* gene cluster, including *pfaA*, *pfaB*, *pfaC*. *pfaD* and *pfaE*, in recombinant cells. Experiments were performed in triplicate.
(PPTX)

S2 Fig Cyclopropane fatty acid, 9,10-methyl-C16:0, was partially degraded after treatment of acidic methylation. A. GC trace of alkaline methylation; B. GC trace of acidic methylation.
(PPTX)

S3 Fig Mass spectra of 3-pyridylcarbinyl 9,10-methylene-hexadecanoate. The distinctive ion that permits location of the ring at delta-9 at *m/z* = 247. The [M-1]^+^ ion (m/z = 358) is more abundant than the molecular ion (*m/z* = 359).
(PPTX)

S4 Fig Evidence of hydroxyl fatty acids in cells. A. FAMEs from DH5α cell harboring *pfaABCDE*. B. Same FAMEs in B derivatized with TMS.
(PPTX)

S5 Fig Mass spectra of 3-hydroxyl fatty acid methyl ester TMS derivatives. A, 3OH-C13:0; B, 3OH-C14:0; C, 3OH-C15:0.
(PPTX)

S1 Table List of primers used in this study. (DOCX)
