# Supplementary material for: DHA Production in Escherichia coli by Expressing Reconstituted Key Genes of Polyketide Synthase Pathway from Marine Bacteria
Source: PLoS One. 2016 Sep 20;11(9):e0162861. doi: 10.1371/journal.pone.0162861 (PMC5029812; doi:10.1371/journal.pone.0162861)
Supplement: S1 Fig — Experiments were performed in triplicate. (PPTX) [file pone.0162861.s001.pptx]

## Slide 1
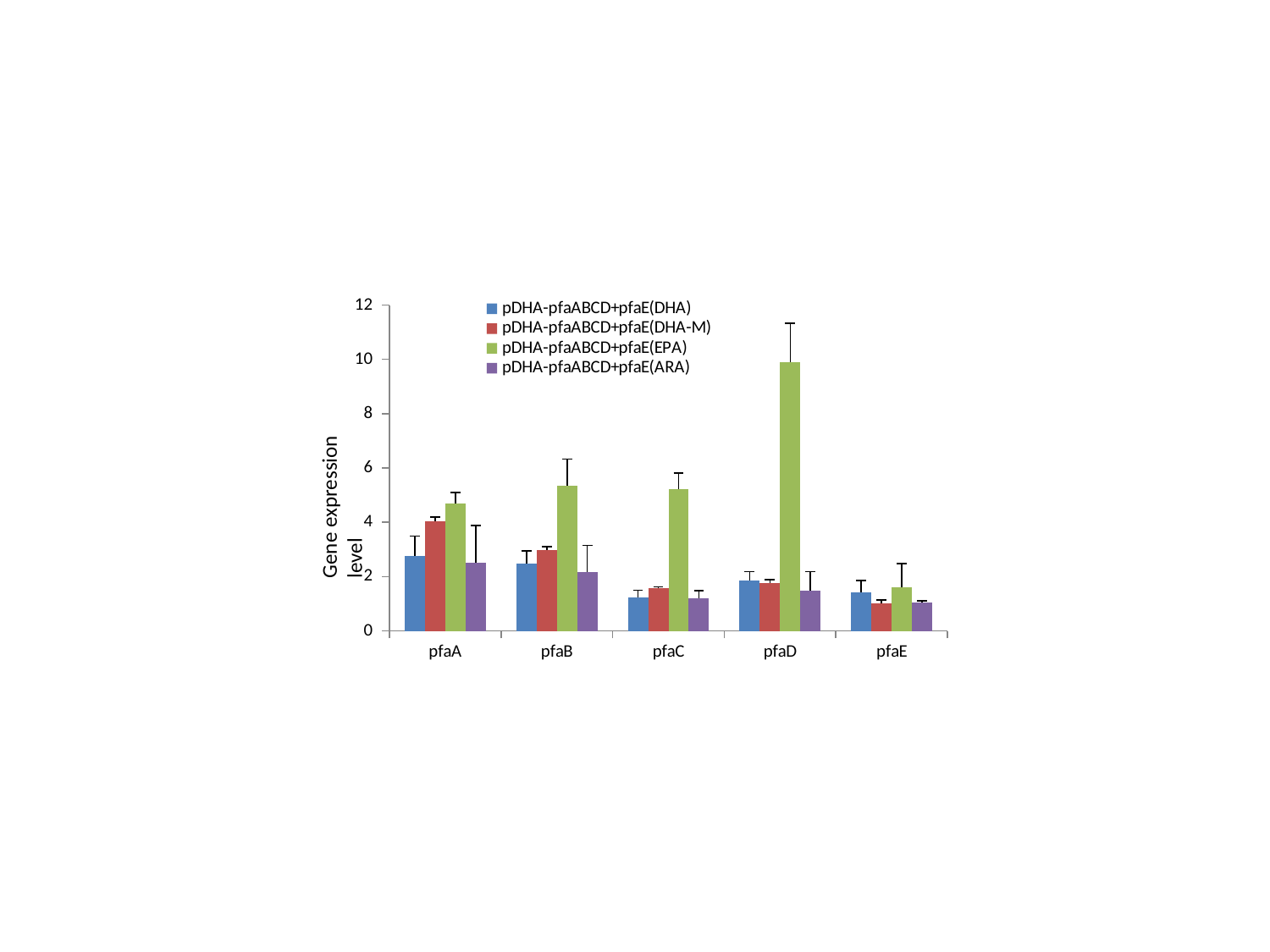

### Chart
| Category | pDHA-pfaABCD+pfaE(DHA) | pDHA-pfaABCD+pfaE(DHA-M) | pDHA-pfaABCD+pfaE(EPA) | pDHA-pfaABCD+pfaE(ARA) |
|---|---|---|---|---|
| pfaA | 2.7534038070833082 | 4.051108348541382 | 4.686477125360567 | 2.5166291507484817 |
| pfaB | 2.469704169007317 | 2.9904931311091607 | 5.359975977209256 | 2.1592656611070726 |
| pfaC | 1.224813404942332 | 1.5665287844950895 | 5.213947603494511 | 1.1982941515182353 |
| pfaD | 1.859584828266082 | 1.7624040185547802 | 9.90904498920355 | 1.4922628747771531 |
| pfaE | 1.4282303655138 | 1.0 | 1.6135308076476877 | 1.04459083316864 |Gene expression level
