# Supplementary figures and images for: DHA Production in Escherichia coli by Expressing Reconstituted Key Genes of Polyketide Synthase Pathway from Marine Bacteria
Source: PLoS One. 2016 Sep 20;11(9):e0162861. doi: 10.1371/journal.pone.0162861 (PMC5029812; doi:10.1371/journal.pone.0162861)

## Slide 1
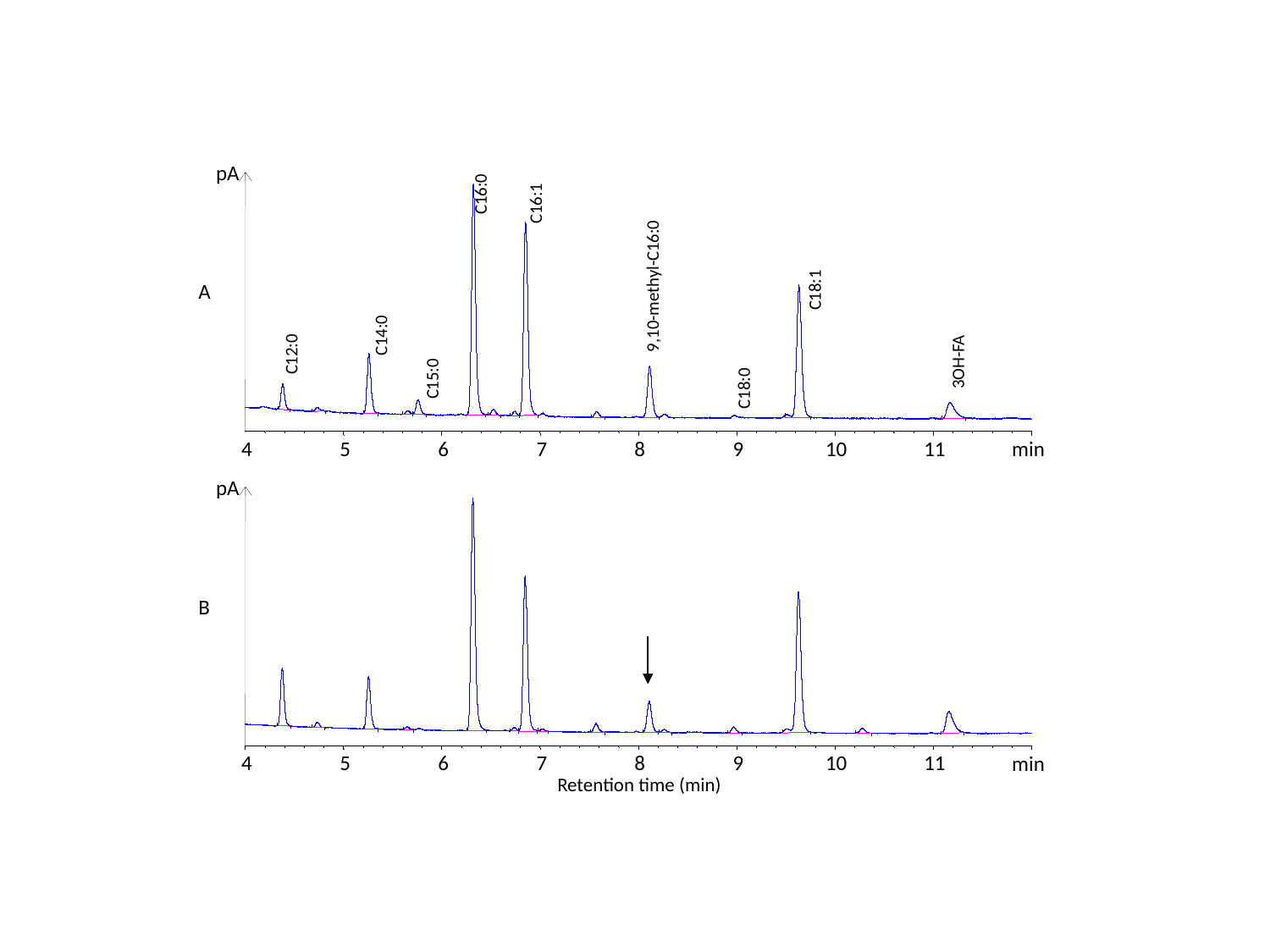

pA
 C16:0
 C16:1
A
 9,10-methyl-C16:0
 C18:1
 C14:0
 C12:0
 3OH-FA
 C15:0
 C18:0
4
5
6
7
8
9
10
11
min
pA
B
4
5
6
7
8
9
10
11
min
Retention time (min)

Supplement: S2 Fig — A. GC trace of alkaline methylation; B. GC trace of acidic methylation. (PPTX) [file pone.0162861.s002.pptx]

## Slide 1
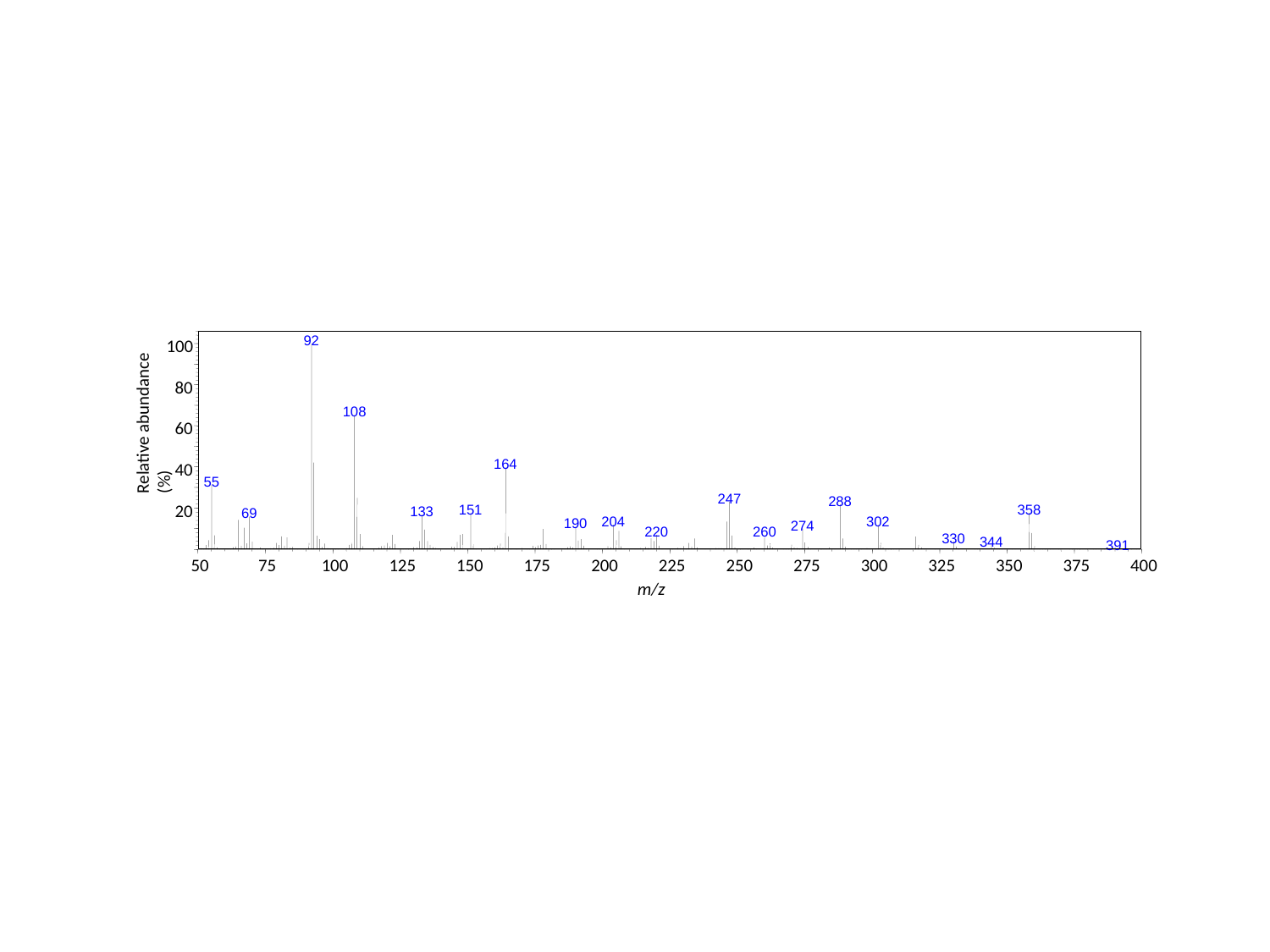

Relative abundance (%)
92
100
80
108
60
164
40
55
247
288
20
358
151
133
69
204
302
190
274
220
260
330
344
391
50
75
100
125
150
175
200
225
250
275
300
325
350
375
400
m/z

Supplement: S3 Fig — The distinctive ion that permits location of the ring at delta-9 at m/z = 247. The [M-1]+ ion (m/z = 358) is more abundant than the molecular ion (m/z = 359). (PPTX) [file pone.0162861.s003.pptx]

## Slide 1
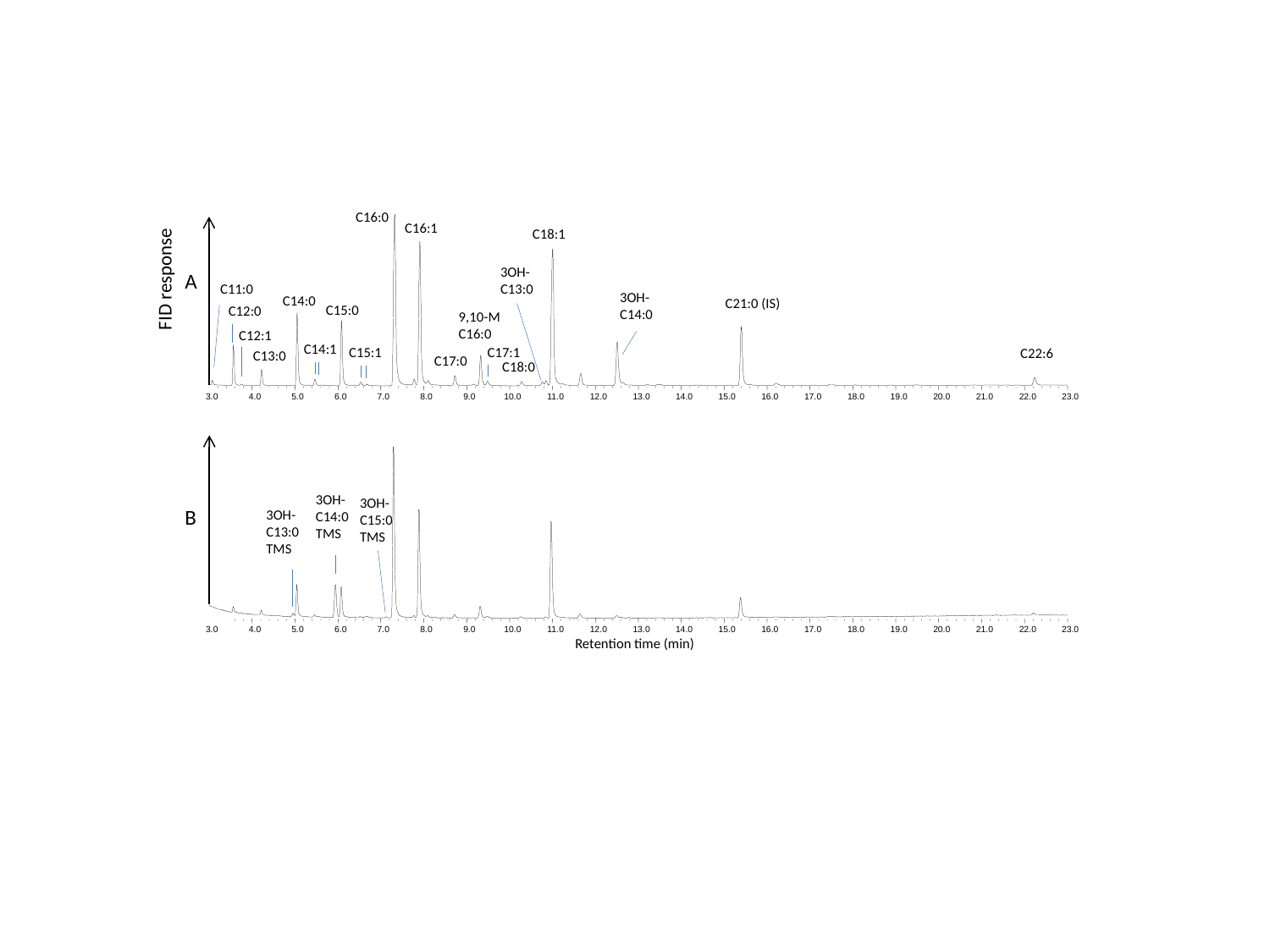

FID response
C16:0
C16:1
C18:1
3OH-
C13:0
A
C11:0
3OH-
C14:0
C14:0
C21:0 (IS)
C15:0
C12:0
9,10-M
C16:0
C12:1
C14:1
C15:1
C17:1
C22:6
C13:0
C17:0
C18:0
3.0
4.0
5.0
6.0
7.0
8.0
9.0
10.0
11.0
12.0
13.0
14.0
15.0
16.0
17.0
18.0
19.0
20.0
21.0
22.0
23.0
3OH-
C14:0
TMS
3OH-
C15:0
TMS
B
3OH-
C13:0
TMS
3.0
4.0
5.0
6.0
7.0
8.0
9.0
10.0
11.0
12.0
13.0
14.0
15.0
16.0
17.0
18.0
19.0
20.0
21.0
22.0
23.0
Retention time (min)

Supplement: S4 Fig — A. FAMEs from DH5α cell harboring pfaABCDE. B. Same FAMEs in B derivatized with TMS. (PPTX) [file pone.0162861.s004.pptx]

## Slide 1
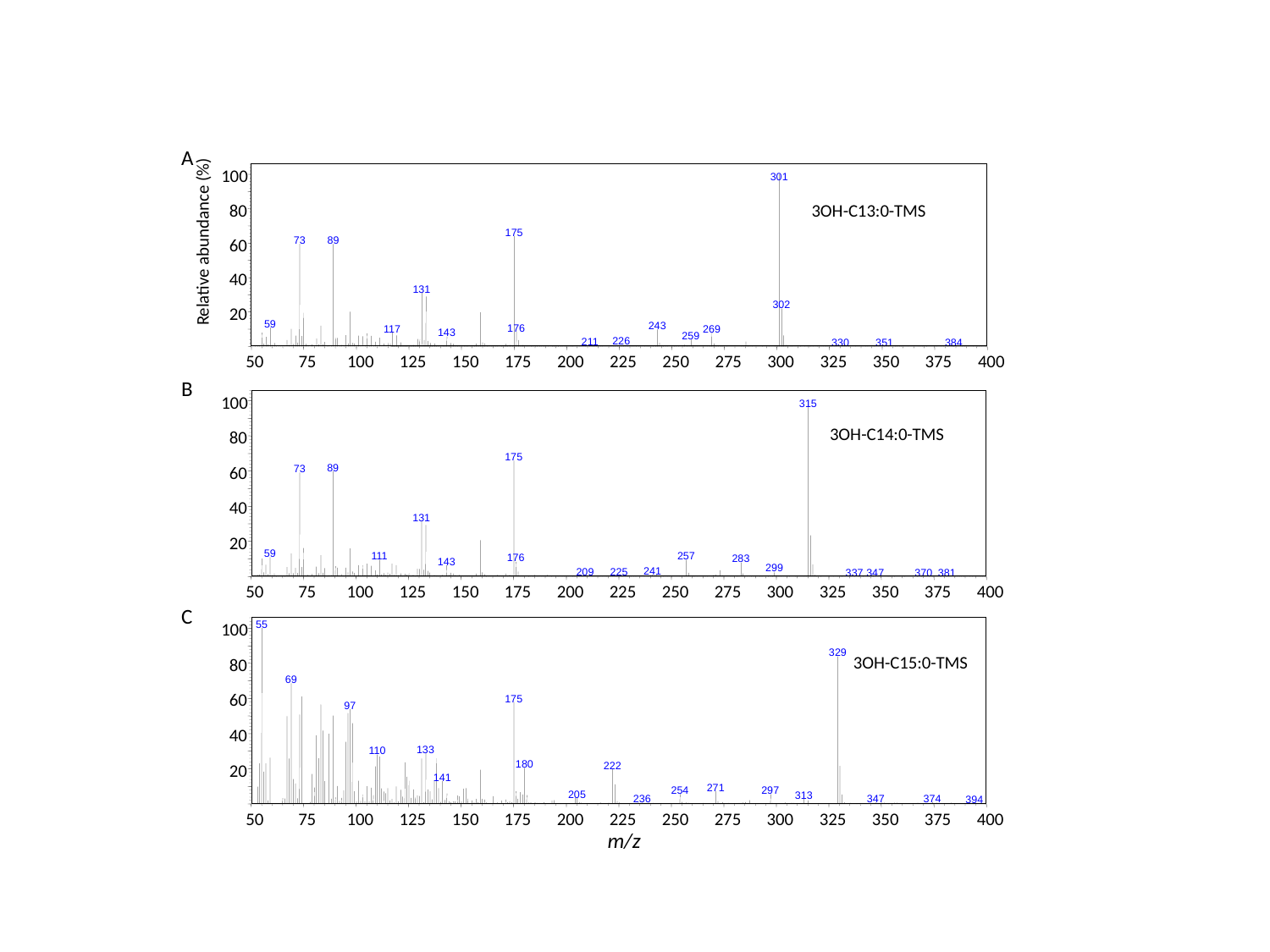

A
Relative abundance (%)
100
301
3OH-C13:0-TMS
80
175
73
89
60
40
131
302
20
59
243
176
117
269
143
259
226
211
330
351
384
50
75
100
125
150
175
200
225
250
275
300
325
350
375
400
B
100
315
3OH-C14:0-TMS
80
175
89
60
73
40
131
20
59
111
257
176
283
143
299
241
209
225
337
347
370
381
50
75
100
125
150
175
200
225
250
275
300
325
350
375
400
C
55
100
3OH-C15:0-TMS
329
80
69
60
175
97
40
133
110
180
222
20
141
271
254
297
205
313
374
236
347
394
50
75
100
125
150
175
200
225
250
275
300
325
350
375
400
m/z

Supplement: S5 Fig — A, 3OH-C13:0; B, 3OH-C14:0; C, 3OH-C15:0. (PPTX) [file pone.0162861.s005.pptx]
