# Supplementary material for: DHA Production in Escherichia coli by Expressing Reconstituted Key Genes of Polyketide Synthase Pathway from Marine Bacteria
Source: PLoS One. 2016 Sep 20;11(9):e0162861. doi: 10.1371/journal.pone.0162861 (PMC5029812; doi:10.1371/journal.pone.0162861)
Supplement: S1 Table — (DOCX) [file pone.0162861.s006.docx]

**Supporting Information**

**Supplementary** **Table 1.** List of primers used in this study.

| Primers | Nucleotide sequence |
| --- | --- |
| pfaA-F | 5’- CG GAATTCATGGCTAAAAAGCAAAGCACATCTAATAACCCTGT-3’ |
| pfaD-R | 5’-ACGC GTCGACCTAATCTTCGCTACGATAGCCAGCCAGTTCAGT-3’ |
| pfaB-F | 5’-ATGGTTAACAATCATTATAAAACGGC-3’ |
| pfaB-R | 5’-TTAAGCTTGTGGCTTTTTCACAATAA-3’ |
| pfaE-1F | 5’- CGG GGTACCATGACTTCTTTTTCTCAATCTGAAC-3’ |
| pfaE-1R | 5’-CGC GGATCCTTAGATTTCCTGATAACCA-3’ |
| pfaE-2F | 5’- CGG GGTACCATGTACAGCGGCGTAAAAGAT-3’ |
| pfaE-2R | 5’-CGC GGATCCCTATTTAGCGTCAGGTTTAA-3’ |
| pfaE-3F | 5’- CGG GGTACCATGATGAACCCAGATA-3’ |
| pfaE-3R | 5’-CGC GGATCCTTAATAATCAGCAAG-3’ |
| pfaE-4F | 5’- CGG GGTACCATGCTTCATTTGCTTTATATCAACT-3’ |
| pfaE-4R | 5’-CGC GGATCCCTAGAATAAAGAATCTACATC-3’ |
| RtA-F | 5’-CTCAGGCAAAGGAAAGCAAC -3’ |
| RtA-R | 5’-CCGTCACTTTGGCAGATTTT-3’ |
| RtB-F | 5’-AGTATGTACGCTGCATTGGG-3’ |
| RtB-R | 5’-CAGCTTGGTCATTGCTAGGG-3’ |
| RtC-F | 5’-TGCTACTGGCACACCAAAAG-3’ |
| RtC-R | 5’-TGAGCCAAGCAAAGGCTTAT-3’ |
| RtD-F | 5’-AGCTACCGGTGATACGGATG-3’ |
| RtD-R | 5’-AGCGTATTTAACGCCATGCT-3’ |
| RtE-1F | 5’-AACCACAAATTTGGCGTAGC-3’ |
| RtE-1R | 5’-CGCTGGTTGCTCTTAACGAT-3’ |
| RtE-2F | 5’-CATGGGGCTTGGGTTTATCG-3’ |
| RtE-2R | 5’-GCCAATGACGCCAAATGTTG-3’ |
| RtE-3F | 5’-TGCGGATTTATCGCCACATG-3’ |
| RtE-3R | 5’-CCATCAAACTGAGCCACACC-3’ |
| RtE-4F | 5’-AACTCTGCTGTGGTTCAAGC-3’ |
| RtE-4R | 5’-CTCCAATTCTTCTGCTGCCC-3’ |
| Rt16S-F | 5’-CCTACGGGAGGCAGCAG-3’ |
| Rt16S-R | 5’-ATTACCGCGGCTGCTGG-3’ |
